# Global sex disparities in lifetime risk of alopecia areata: a systematic analysis from the global burden of disease study, 1990 to 2021

**DOI:** 10.1186/s13293-025-00749-w

**Published:** 2025-09-24

**Authors:** Jiachen Sun, Yuhao Li, Zhenzhen Ye, Shengfeng Wang, Wenhui Wang

**Affiliations:** 1 Department of Dermatology, Peking University Third Hospital, Beijing, China; 2 Department of Epidemiology and Biostatistics, School of Public Health, Key Laboratory of Epidemiology of Major Diseases, Peking University, Ministry of Education, Beijing, China; 3 Institute for Artificial Intelligence, Peking University, Beijing, China

**Keywords:** Alopecia areata, Sex disparity, Global burden of disease, Lifetime risk

## Abstract

**Background:**

Alopecia areata (AA) represents a significant autoimmune disorder affecting hair follicles, with sex differences that have yet to reach scientific consensus. The regional and temporal, age-related, and socioeconomic dimensions of sex-based lifetime risk disparities remain inadequately characterized and require further investigation.

**Methods:**

(1) We estimated AA lifetime risk using the adjusted for multiple primaries method. We evaluated regional and temporal features of sex disparities through Average Annual Percentage Change analysis, with future projections to 2050 via ARIMA modeling. (2) Age-stratified sex disparity features were analyzed. (3) We evaluated AA risk associations with Socio-Demographic Index (SDI) variations through Spearman correlation analysis and concentration indices to quantify female-to-male relative risk.

**Results:**

(1) Global lifetime risk increased from 29.89% (95% CI, 29.83%-29.94%) to 31.66% (31.61%-31.71%) in females, and from 15.91% (15.86%-15.95%) to 16.93% (16.88%-16.97%) in males between 1990 and 2021. The female-to-male lifetime risk ratio remained stable, measuring 1.88 (1.87–1.89) in 1990 and 1.87 (1.86–1.88) in 2021. Future projections through 2050 indicate increasing lifetime risks reaching 34.44% (32.99%-35.89%) for females and 19.06% (17.50%-20.63%) for males, despite comparatively faster growth rates observed in the male population (9.83% vs. 7.01% for females). Both sexes exhibited similar regional distribution features. (2) Sex differences in age-specific risk features were notable: females exhibited a high lifetime risk window between ages 20–50 years, while males demonstrated a narrower window primarily between ages 20–30 years. Female lifetime risk consistently peaked in the 30–39 age group regardless of SDI level, whereas males displayed significant SDI-dependent variations, with peak lifetime risks occurring at ages 20–29 in high-SDI regions versus ages 30–39 in the other areas. Females showed a slower decline in residual risk with increasing age compared to males, resulting in progressively higher female-to-male risk ratios that were particularly pronounced in high-SDI regions, reaching 4.51 (3.42–5.95) in the 70–79 age cohort. (3) With increasing SDI levels (reflecting socioeconomic development), females exhibited more pronounced risk elevation than males. Females exhibited consistently lower concentration indices relative to males, maintaining stable trends, while male concentration indices have shown a declining trend in recent years. The female-to-male ratio of concentration indices has shown a consistent upward trend since reaching its lowest point of 0.62 (0.51–0.76) in 1993, rising to 0.75 (0.60–0.95) by 2021. High SDI regions persistently demonstrate the lowest female-to-male concentration indices.

**Conclusions:**

Our analysis reveals pronounced sex disparities in global AA lifetime risk: consistently higher risk in females across regions and time periods, prolonged age-related risk window with slower decline in females, and greater risk elevation in females with increasing SDI levels.

**Supplementary Information:**

The online version contains supplementary material available at 10.1186/s13293-025-00749-w.

## Highlights

Our analysis reveals pronounced sex disparities in global AA lifetime risk: consistently higher risk in females across regions and time periods, prolonged age-related risk window with slower decline in females, and greater risk elevation in females with increasing SDI levels.

Females consistently demonstrated higher lifetime risk of AA than males globally, with the female-to-male ratio remaining stable at approximately 1.9 throughout 1990–2021.

Females maintained elevated risk across a broader age range (20–50 years) compared to males (20–30 years), with females showing a slower decline in residual risk with increasing age compared to males.

With increasing SDI levels (reflecting socioeconomic development), females exhibited more pronounced risk elevation than males.

**Supplementary Information:**

The online version contains supplementary material available at 10.1186/s13293-025-00749-w.

## Abstract (plain language summary)

Hair loss from alopecia areata affects millions worldwide, with female and males experiencing this condition differently. Our study examined these sex differences across countries, age groups, and socioeconomic levels from 1990 to 2021, with projections to 2050. We found that females consistently face a higher lifetime risk of developing alopecia areata compared to males—about 1.9 times higher. By 2021, nearly 32% of females and 17% of males worldwide were at risk of developing this condition during their lifetime, with these numbers expected to increase to 34% for females and 19% for males by 2050.

Analysis of sex disparities in AA lifetime risk revealed: (1) Females consistently had higher lifetime risk than males globally, with this 1.9-fold difference remaining stable over three decades; (2) Females maintained elevated risk across a broader age range (20–50 years) compared to males (20–30 years), with females showing a slower decline in residual risk with increasing age compared to males; (3) With socioeconomic development, females exhibited more pronounced risk elevation than males.

These findings highlight the need for sex-specific approaches to alopecia areata prevention, diagnosis, and treatment, taking into account how social and economic factors influence these sex disparity across the lifespan.

**Supplementary Information:**

The online version contains supplementary material available at 10.1186/s13293-025-00749-w.

## Introduction

The increasing incidence of autoimmune disorders in recent decades has emerged as a significant global public health concern [1]. Alopecia areata (AA) is a follicle-specific autoimmune disease characterized by patchy hair loss, resulting from the combined effects of genetic and environmental factors [2]. AA affects approximately 0.2% of the global population, with distinct demographic and geographic distribution features [3]. The visible manifestation of AA significantly impacts patients’ appearance, potentially leading to psychological distress, with approximately 30–68% of AA patients experiencing varying degrees of depression and anxiety [4]. Notably, epidemiological evidence regarding sex disparities in AA risk profiles has yielded conflicting results. While some studies suggest female predominance in AA incidence [5], others have documented comparable rates between sexes [6, 7]. This inconsistency highlights a critical knowledge gap in our understanding of sex disparities in AA epidemiology, particularly concerning regional and temporal, age-related, and socioeconomic dimensions.

Lifetime risk represents an optimal metric for evaluating the cumulative sex-specific AA burden, yet remains underutilized in existing AA research literature. The lifetime risk of developing AA quantifies an individual’s probability of developing this condition throughout their complete lifespan trajectory (from birth to death) [8, 9]. This epidemiological measure provides a longitudinal perspective on the cumulative probability of AA development while accounting for demographic transitions and competing mortality risks [10, 11]. When integrated with complementary epidemiological indicators, lifetime risk analysis enhances comprehensive interpretation of AA’s global epidemiological profile.

The Global Burden of Disease (GBD) study provides a comprehensive framework for analyzing disease features across more than 200 countries and regions [12]. Leveraging this resource, our study investigated sex disparities in global AA lifetime risk from 1990 to 2021, with projections through 2050. Through comprehensive analysis of sex disparity across regional and temporal, age-related, and socioeconomic dimensions, we aim to provide evidence-based insights for targeted intervention strategies, resource allocation, and future research directions for AA management globally.

## Methods

### Data sources

The detailed methodology for GBD data collection and processing has been previously described [13, 14]. Downstream data analysis employs three statistical models: meta-regression-Bayesian regularized trimmed (MR-BRT), a statistical method that combines meta-regression techniques with Bayesian approaches for bias adjustment and uncertainty quantification; DisMod-MR 2.1 (Disease Modeling-Meta Regression version 2.1), a compartmental modeling framework designed for epidemiological data analysis; and spatiotemporal Gaussian process regression (ST-GPR), a modeling technique that captures spatial and temporal correlations to generate smooth estimates across different geographic locations and time periods. All disease terminology was standardized using International Classification of Disease (ICD) codes to ensure accuracy and comparability. The study adheres to the Guidelines for Accurate and Transparent Health Estimates Reporting (GATHER) [15].

We extracted age-, sex-, and location-specific incidence data for AA from 1990 to 2021, along with total population and all-cause mortality data to account for competing risks in lifetime risk calculations. This study did not involve any personal or sensitive information; therefore, ethical approval was not required.

Additionally, we utilized the Socio-Demographic Index (SDI), a composite indicator developed by the GBD Study Collaborators to measure the overall development level of countries and regions [16]. The methodological framework for calculating SDI was first introduced in the 2016 Lancet publications of the GBD Study 2015, adopting the computational principles underlying the Human Development Index to establish a comprehensible and standardized scale [17]. The SDI is calculated as the geometric mean of three standardized components: (1) total fertility rate among females under 25 years of age, (2) mean educational attainment in the population aged 15 years and older, and (3) lag-distributed income per capita. The SDI values range from 0 to 1, where higher values indicate higher socio-demographic development. Countries are typically categorized into five SDI quintiles: low SDI (≤ 0.46), low-middle SDI (0.46–0.60), middle SDI (0.60–0.69), high-middle SDI (0.69–0.81), and high SDI (> 0.81), enabling comparative analysis of disease burden across different development levels.

### Disease definition

During the GBD study, AA was included as a cause of skin and subcutaneous conditions and identified using standardized diagnostic codes following the methodology established by the GBD Study Collaborators [18]. The case definition requires clinical diagnosis consistent with specific ICD codes and excludes other forms of hair loss such as androgenetic alopecia or drug-induced alopecia. The ICD-10 classification system, maintained by the World Health Organization (WHO) as the international standard for diagnostic classification [19], uses code L63 to encompass “Alopecia areata” with specific subcategories: L63.0 (Alopecia totalis), L63.1 (Alopecia universalis), L63.2 (Ophiasis), L63.8 (Other alopecia areata), and L63.9 (Alopecia areata, unspecified). The corresponding ICD-9 code is 704.01 [20]. These ICD codes for AA identification have demonstrated high diagnostic accuracy with a positive predictive value of 88.9% in validation studies [21], supporting their reliability for case identification in GBD analyses.

### Statistical analysis

#### Lifetime risk

We employed the adjusted for multiple primaries method to estimate the lifetime risk of AA for the total population, males, and females across 204 countries/territories globally [9–11]. This method accounts for competing mortality and adjusts for the impact of multiple primary events included in incidence rates. We used age-specific incidence rates and all-cause mortality data stratified by 5-year age groups to calculate the lifetime risk of developing AA for both males and females across different age groups.

To quantify sex-based differences in AA lifetime risk, we calculated female-to-male relative risk (RR) ratios and their corresponding 95% confidence intervals (CIs). The analysis assumed uniform distribution of lifetime risk estimates within their respective 95% CIs, which represents a conservative approach for uncertainty propagation. Relative risk was computed as the ratio of female lifetime risk to male lifetime risk (RR = Risk_female / Risk_male), where values greater than 1.0 indicate higher risk in females, and values less than 1.0 indicate higher risk in males. To account for uncertainty in both numerator and denominator, we employed a Monte Carlo simulation approach with 100,000 resampling iterations. In each iteration, random values were drawn from the uniform distributions of female and male lifetime risks within their respective CIs, and the RR was calculated. This resampling procedure generated an empirical distribution of RR values, from which we derived the 95% CI by identifying the 2.5th and 97.5th percentiles. This bootstrap-based approach provides robust estimates of sex disparities while appropriately propagating the uncertainty inherent in the original lifetime risk estimates.

#### Average annual percentage change

Temporal trends in lifetime risk were assessed using the Average Annual Percentage Change (AAPC) and its 95% confidence interval (CI), calculated using Joinpoint trend analysis software (command line version 5.3.0) [22]. The AAPC is a summary measure that describes the average rate of change over a specified time period, accounting for potential changes in trend direction. Joinpoint regression analysis identifies points in time where statistically significant changes in trends occur by fitting a series of joined straight lines to the data on a log scale [23, 24]. The software uses a grid search method to identify the optimal number and location of joinpoints that best fit the data, with statistical significance determined by Monte Carlo permutation tests. This approach allows for the detection of complex temporal features and provides robust estimates of trend changes, making it particularly suitable for analyzing long-term epidemiological data where trend directions may vary over time. Negative AAPC indicates a declining trend, while positive AAPC indicates an increasing trend.

#### ARIMA modeling

We utilized Autoregressive Integrated Moving Average (ARIMA) models to forecast AA lifetime risk from 2021 to 2050 [25]. ARIMA is a statistical forecasting method that combines three components: autoregression (AR), which uses past values to predict future values; integration (I), which accounts for non-stationarity by differencing the time series data; and moving average (MA), which incorporates past forecast errors to improve predictions. Model selection was based on autocorrelation function (ACF) and partial autocorrelation function (PACF) analyses to determine the optimal orders of autoregression (p), differencing (d), and moving average (q) parameters, denoted as ARIMA(p, d,q). The ACF measures the correlation between observations at different time lags, while PACF measures the correlation between observations after removing the effects of intervening observations. Model optimization utilized multiple statistical criteria: Akaike Information Criterion (AIC), which balances model fit and complexity by penalizing the number of parameters; Bayesian Information Criterion (BIC), which applies a stronger penalty for model complexity than AIC; and Root Mean Square Error (RMSE), which measures the average magnitude of prediction errors [26, 27]. The model with the lowest combination of these criteria was selected as the optimal forecasting model, ensuring both accuracy and parsimony in our projections.

#### Concentration index

We employed concentration indices to represent inequities in AA burden across countries with different development levels based on SDI. The concentration index ranges from − 1 to 1, with negative values indicating burden concentration in low-SDI regions and positive values indicating concentration in high-SDI regions [28]. A concentration index of zero indicates perfect equality. The concentration index was calculated using the formula: CI = (2/µ) × Cov(h, R), where µ is the mean health outcome, h represents the AA burden for each country, and R is the fractional rank of countries ordered by SDI. The covariance between health outcomes and SDI rankings provides a standardized measure of inequality that accounts for both the magnitude of health disparities and the distribution of countries across development levels. To assess the statistical significance of observed inequalities, we calculated 95% CIs for concentration indices using bootstrap resampling with 100,000 iterations. Concentration indices were interpreted as follows: values between − 0.2 and 0.2 indicated relatively low inequality, values between ± 0.2 and ± 0.5 indicated moderate inequality, and values beyond ± 0.5 indicated high inequality in AA burden distribution across SDI levels.

Additionally, we calculated the female-to-male ratio of concentration indices across different SDI regions and time periods to quantify sex-specific features of socioeconomic inequality in AA burden. This ratio was computed by dividing the female concentration index by the male concentration index for each year and SDI region. The 95% confidence intervals for these ratios were also calculated using bootstrap resampling methods to assess the statistical significance of observed sex differences in socioeconomic-related AA inequalities.

All analyses were conducted with SAS 9.4 and R 4.4.2. Statistical significance was defined as *P* < 0.05 using two-sided tests.

## Results

### Overall sex disparities in global AA lifetime risk

**Fig. 1:**
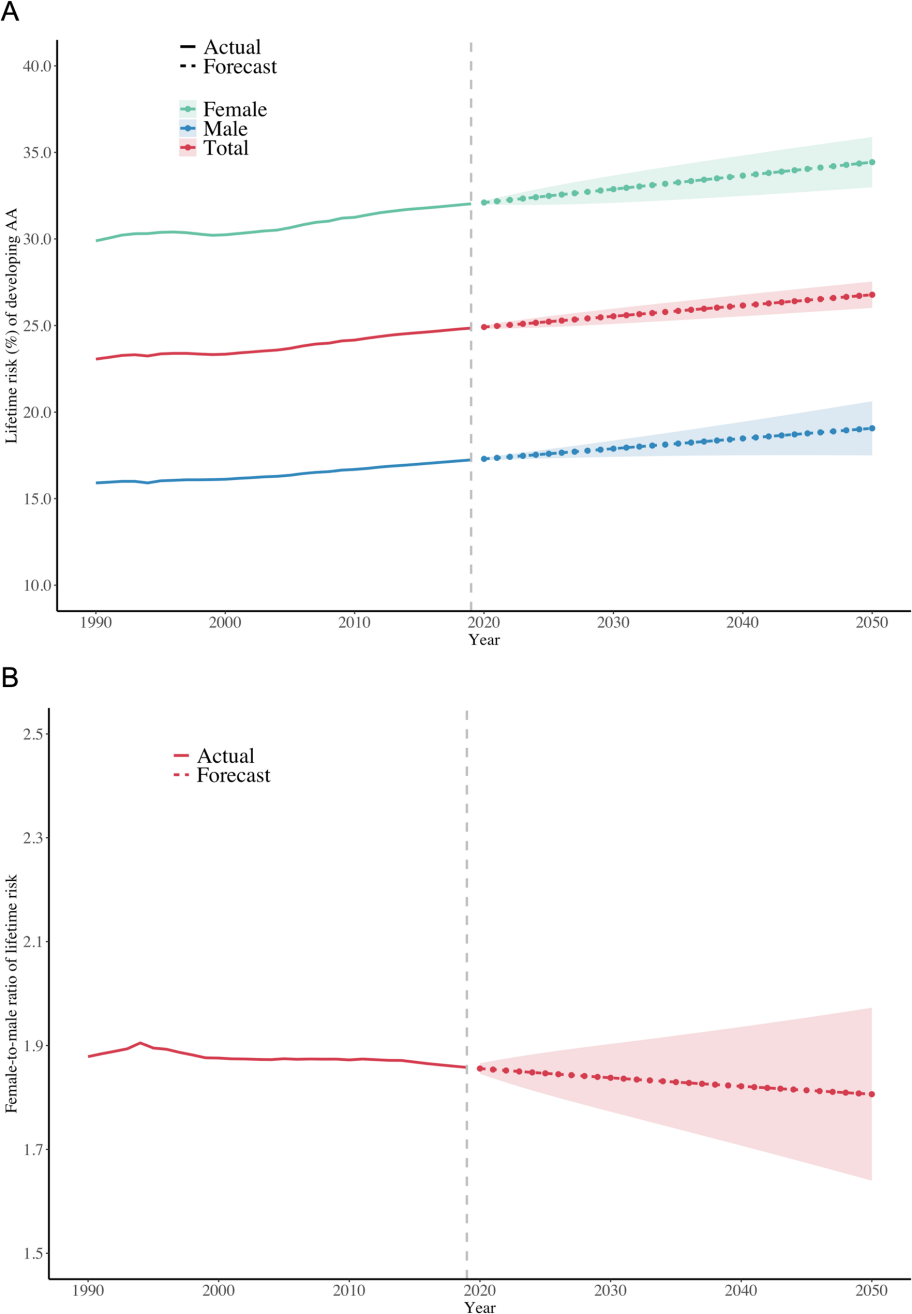
Projected global AA lifetime risk trends stratified by sex (**A**) and corresponding female-to-male lifetime risk ratio (**B**), 1990–2050

Our analysis reveals a persistent and substantial female predominance in AA susceptibility globally, with this sex disparity remaining remarkably stable over three decades despite differential growth features between sexes. The global lifetime risk (Table S1, Fig. 1A) of females increased from 29.89% (95% CI, 29.83%-29.94%) in 1990 to 31.66% (31.61%-31.71%) in 2021. For males, the lifetime risk rose from 15.91% (15.86%-15.95%) in 1990 to 16.93% (16.88%-16.97%) in 2021. The female-to-male lifetime risk ratio remained stable, measuring 1.88 (1.87–1.89) in 1990 and 1.87 (1.86–1.88) in 2021 (Table S1). Future projections through 2050 indicate increasing lifetime risks reaching 34.44% (32.99%-35.89%) for females and 19.06% (17.50%-20.63%) for males (Fig. 1A, Table S2), despite comparatively faster growth rates observed in the male population (9.83% vs. 7.01% for females). The female-to-male ratio of lifetime risk exhibited a marginal decrease from 1.85 (1.84–1.87) in 2021 to 1.81 (1.64–1.97) in 2050, indicating persistent substantial female predominance throughout the projection period (Fig. 1B, Table S2). This persistence despite faster male growth rates suggests that intrinsic biological factors, rather than temporal environmental changes, may be the primary drivers of sex-based susceptibility differences in AA.

### Regional and temporal variations in sex disparities

**Fig. 2:**
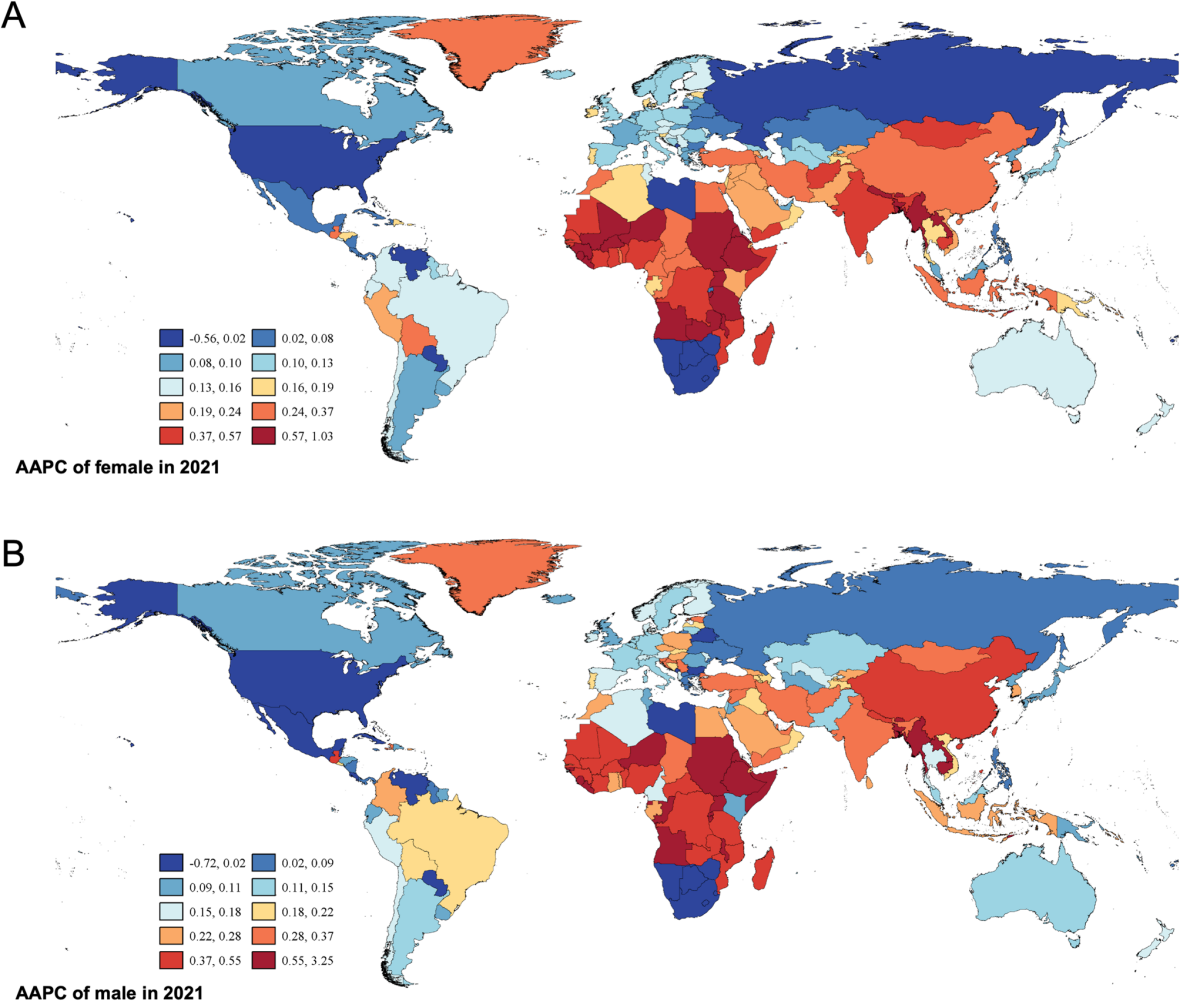
AAPC of lifetime risk for females (**A**) and males (**B**) during 1990–2021

Building upon the observed global sex disparities, we examined regional features and temporal trends to understand the geographic distribution of AA burden. Geographic analysis revealed substantial regional variations in lifetime risk features. In 1990 (Fig. S1A), significantly higher lifetime risks were observed in North America, Western Europe, Southern Latin America, and high-income Asia-Pacific regions compared to other global areas. This feature persisted through 2021 (Fig. S1B), with notable increases in China and Southeast Asian regions. Importantly, females consistently demonstrated higher lifetime risk across virtually all geographic regions (Fig. S2, S3). Global AAPC was estimated at 0.186% (0.143%-0.229%) for females and 0.201% (0.165%-0.230%) for males, indicating that males demonstrated a relatively higher rate of annual increase in disease burden globally (Fig. 2).

Temporal trend analysis from 1990 to 2021 (Fig. S4) revealed a consistent feature where high-income regions maintained elevated lifetime risks despite lower AAPCs, while low-income regions demonstrated lower absolute risks but higher AAPCs, indicating accelerated growth rates (Table S3). Regional analysis by sex showed distinct features: females consistently demonstrated the highest risk in high-income North America, high-income Asia-Pacific, and Australasia throughout the study period (Fig. S5), while males exhibited the highest lifetime risk in Western Europe, high-income Asia-Pacific, and Australasia (Fig. S6).

### Age-related variations in sex disparities

**Fig. 3:**
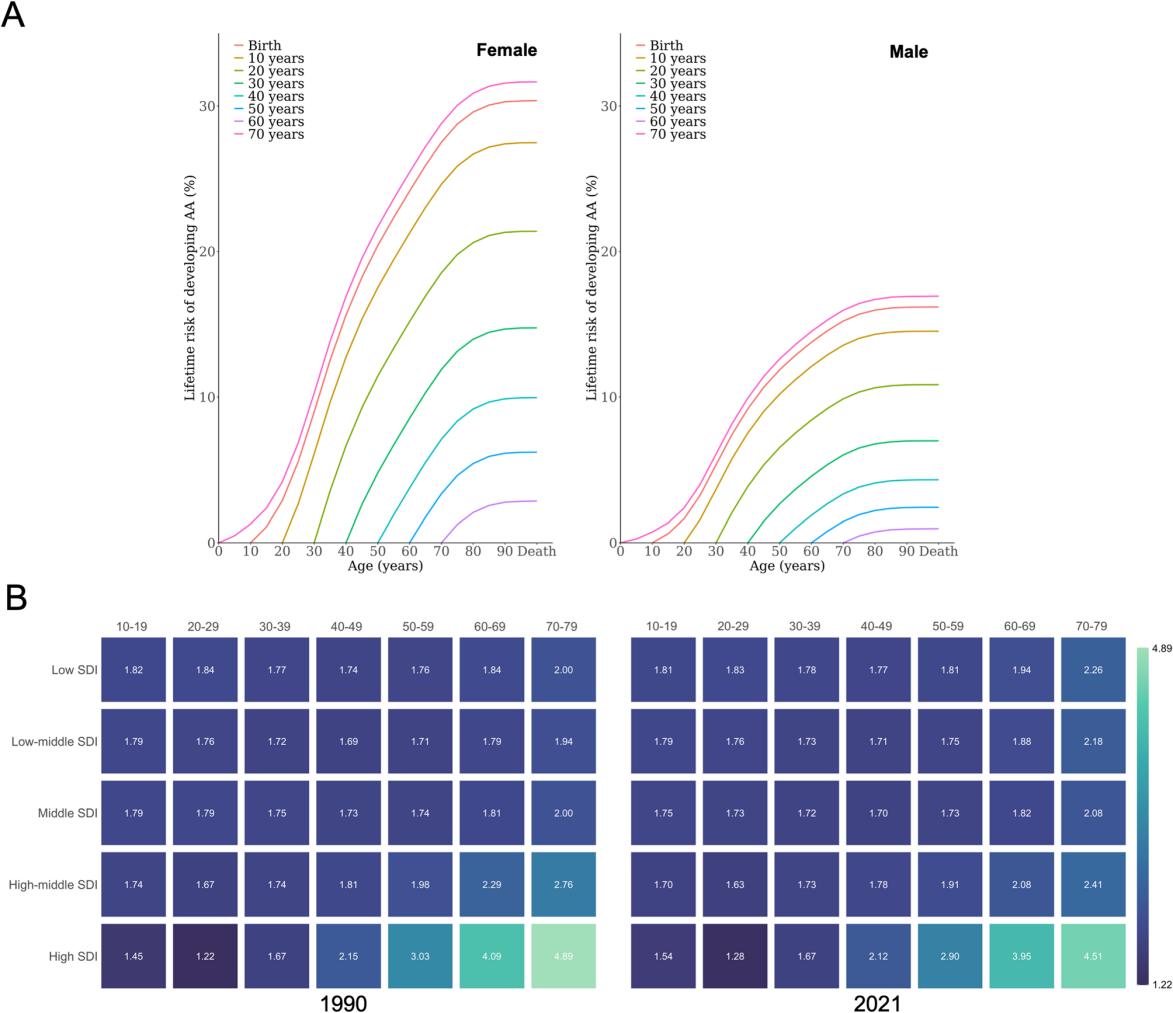
Age-stratified lifetime risks in 2021 by sex (**A**). Female-to-male relative risk across age groups by SDI levels (**B**)

Having confirmed the overall elevation in female lifetime risk, we next examined how these features manifest across different age groups. Females exhibited a characteristic feature of increasing risk from ages 20–50 years (Fig. 3A, Table S4), with peak lifetime risk occurring in the 30–39 age group across all SDI levels. In contrast, males demonstrated earlier peak risk occurrence, primarily between ages 20–30 years, with notable regional heterogeneity: high-SDI regions showed peak risks at ages 20–29, while other regions peaked at ages 30–39.

SDI-dependent analysis (Fig. S7) further revealed how socioeconomic development shapes these age-specific features. Among females, the 30–39 age group consistently showed the highest lifetime risk across all SDI levels, but the risk levels varied greatly among different SDI regions. The high-to-low SDI ratio in this peak age group decreased from 1.76 (8.45%/4.79%) in 1990 to 1.42 (8.09%/5.71%) in 2021, reflecting narrowing yet persistent disparities. Importantly, post-peak trajectories differed markedly by SDI level: less developed regions showed rapid risk decline after age 39, while high-SDI areas maintained substantial risk (> 5%) through ages 60–69 in 2021, highlighting how socioeconomic factors extend AA risk into older age groups among females. In contrast, males exhibited more uniform post-peak features, with all SDI regions showing steep lifetime risk decline after age 39. This suggests that socioeconomic factors may have less influence on extending male AA risk into later age groups compared to females.

The female-to-male relative risk analysis (Fig. 3B) provided additional insights into how sex disparities evolve across the lifespan and development levels. In low-SDI areas, female-to-male relative risk remained relatively stable across age groups. However, with increasing SDI levels, a pronounced age-dependent feature emerged. High-SDI regions displayed lower relative risk in younger cohorts (10–39 years) compared to other regions, but demonstrated substantially elevated relative risk in older age groups (40–79 years). Most strikingly, in high-SDI regions, the relative risk among individuals aged 70–79 years reached 4.89 in 1990 and, despite subsequent reduction, remained markedly high at 4.51 in 2021. This feature underscores how socioeconomic development not only increases overall female AA susceptibility, but also amplifies sex disparities in elderly populations, potentially reflecting cumulative effects of differential healthcare access, lifestyle factors, and hormonal influences across the lifespan.

### SDI-related variations in sex disparities

**Fig. 4:**
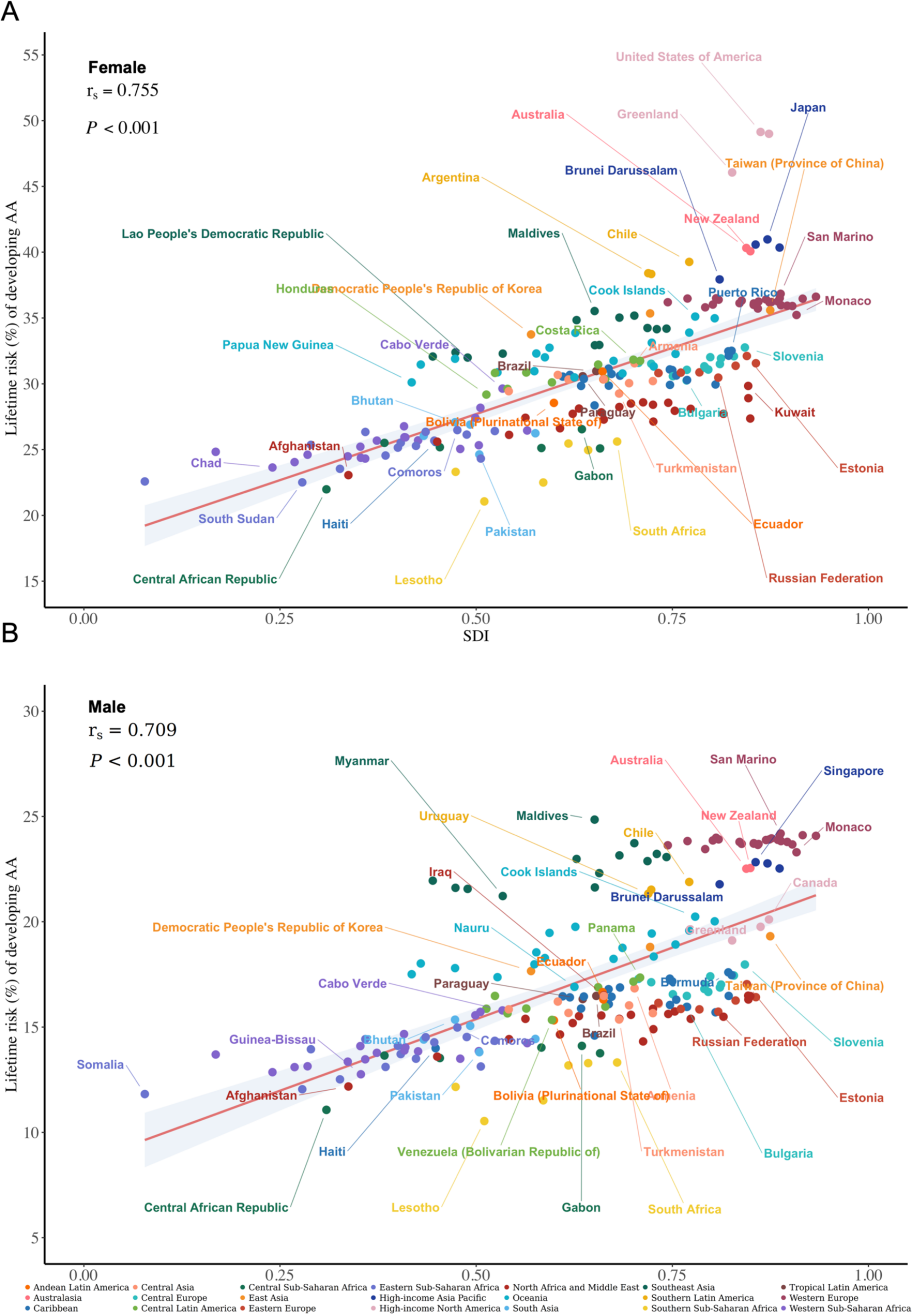
SDI correlation analysis with lifetime risk for females (**A**) and males (**B**). Countries are classified and color-coded according to 21 geographical regions based on the GBD regional classification. Each region displays two representative countries for the respective sex: those with the highest and lowest lifetime risks within the region, illustrating intra-regional health disparities and facilitating comparison of variation ranges

We examined the relationship between socioeconomic development and sex disparities in AA lifetime risk, hypothesizing that development levels might differentially impact male and female AA features. Figure 4 presents the correlation analysis between SDI and AA lifetime risk for both sexes, with countries color-coded by 21 geographical regions. Each region displays two representative countries for the respective sex: those with the highest and lowest lifetime risks within the region. The analysis revealed a key finding: females exhibited a stronger positive correlation between SDI and AA lifetime risk compared to males (females *rs* = 0.755, males *rs* = 0.709), with sex-specific correlation features resulting in different labeled countries between females and males, reflecting distinct regional disparity features.

**Fig. 5:**
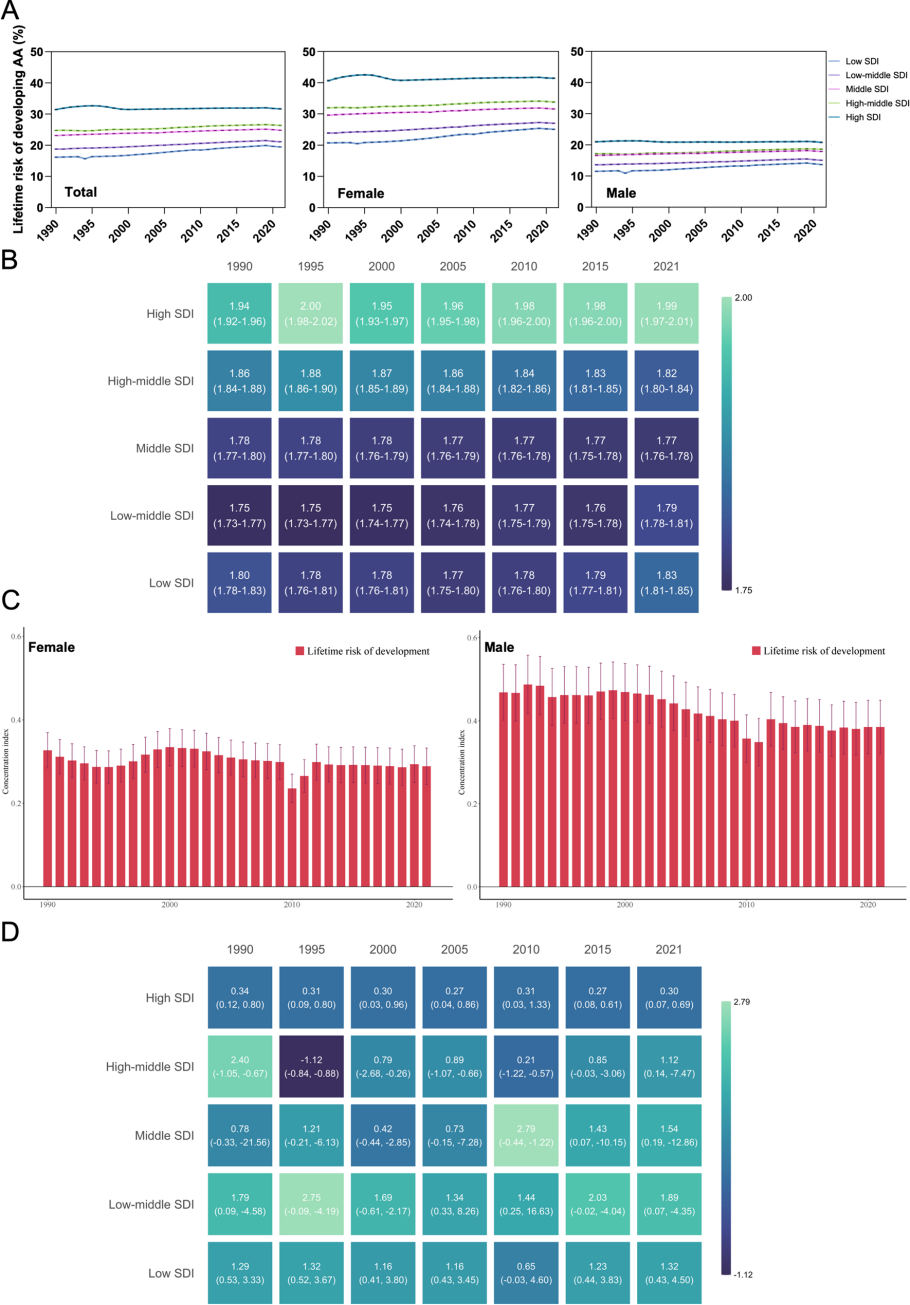
SDI-stratified analysis of AA lifetime risk by sex (**A**), and heatmap of female-to-male lifetime risk ratio across SDI regions (**B**). Concentration index trends by sex (**C**) and heatmap of female-to-male ratio across SDI regions (**D**)

This differential relationship was further confirmed by SDI-stratified analysis (Fig. 5A), which revealed more pronounced disparities across SDI levels among females. In 1990, the lifetime risk disparity between high and low SDI regions was 1.96-fold for females (40.66% versus 20.74%), which decreased slightly to 1.65-fold (41.45% versus 25.09%) in 2021. In contrast, males demonstrated a 1.83-fold difference (20.99% versus 11.50%) in 1990, decreasing to 1.52-fold (20.82% versus 13.70%) by 2021. Consistent with these features, analysis of female-to-male lifetime risk ratios across different SDI regions revealed that high-SDI regions consistently demonstrated the highest ratio values (Fig. 5B, Table S5). Collectively, these findings suggest that socioeconomic development disproportionately influences female AA susceptibility, particularly at higher SDI levels, potentially through differential healthcare-seeking behaviors, diagnostic access, or lifestyle factors associated with economic development.

To further understand how socioeconomic factors shape sex-based health inequalities in AA, we examined the concentration index, which quantifies the degree to which disease burden is concentrated among higher socioeconomic groups. Analysis of the concentration index (Fig. 5C) revealed distinct temporal features between sexes. Among females, the concentration index reached its peak in 2000 (0.334, 95% CI 0.290–0.379), followed by a gradual, steady decline to 0.289 (0.245–0.332) by 2021. Males exhibited comparatively higher concentration indices overall, with peak values observed in 1999 (0.474, 95% CI 0.406–0.541), followed by a more pronounced downward trajectory, reaching 0.385 (0.321–0.449) by 2021. The female-to-male concentration index ratio increased from 0.70 (0.57–0.85) in 1990 to 0.75 (0.60–0.95) in 2021 (Table S6), indicating persistent sex-based disparities throughout the three-decade period, with males consistently showing higher concentration of AA burden among affluent populations.

Analysis of female-to-male concentration index ratios across different SDI regions (Fig. 5D) further illuminated these disparities. High-SDI regions consistently exhibited the lowest ratios, maintaining remarkable stability (~ 0.30) throughout the study period. In contrast, low- and low-middle SDI regions demonstrated persistently higher ratios, suggesting that females in these areas experienced greater socioeconomic-related health inequalities than males. These SDI-related variations reflect systematic differences in sex equality, healthcare accessibility, and socioeconomic determinants of health across development level.

## Discussion

This study provides the first comprehensive analysis of sex disparities in AA lifetime risk using three decades of global data, revealing consistent female predominance across temporal and geographic, age-specific, and socioeconomic dimensions. Our analysis identified three sex disparity features: (1) Females consistently demonstrated higher lifetime risk of AA than males globally, with the female-to-male ratio remaining stable at approximately 1.9 throughout 1990–2021; (2) Females maintained elevated risk across a broader age range (20–50 years) compared to males (20–30 years), with females showing a slower decline in residual risk with increasing age compared to males; (3) With socioeconomic development, females exhibited more pronounced risk elevation than males.

Given the persistently increasing lifetime risk of AA in both females and males, our research necessitates a paradigm shift in viewing AA from a relatively underrecognized dermatological condition to a global health equity issue with high lifetime risk. The elevated lifetime risk of AA in females may result from the complex interplay of biological, socioculturally, and socioeconomic developmental factors. Previous studies have documented female predominance in AA across diverse populations [29–33]. Biologically, estradiol modulation of hair follicle cycling [34], estrogen enhancement of T-cell autoimmunity [35, 36], and X-chromosome-linked immune gene escape [37, 38] provide plausible mechanisms for female susceptibility. Socioculturally, females may be disproportionately affected due to heightened aesthetic concerns, increased healthcare-seeking behavior, greater exposure to hair styling chemicals, and enhanced stigmatization of female hair loss compared to socially accepted male baldness. Socioeconomically, high-SDI related lifestyles may further exacerbate the biological and social vulnerabilities contributing to female AA susceptibility, including higher obesity prevalence among females (11.5% vs. 6.9% in severe obesity) [39, 40], intensified appearance-related social pressures (68.5% vs. 1.5% high appearance concern in Australian females vs. males) [41], and differential stress exposure during extended reproductive years. The interplay of these factors may contribute to the elevated and persistently increasing lifetime risk of AA in females.

From regional and temporal features of sex disparity, the elevated and persistently increasing lifetime risk of AA in females indicates that current prevention, treatment, and health education strategies remain inadequate. For high-income countries and regions, the continued albeit modest increase in AA lifetime risk suggests that better prevention strategies should focus on risk factors associated with high-income settings, including stress management [42, 43], controlling exposure to hair styling chemicals [44, 45], and targeted healthcare approaches for females who are more affected [46, 47]. For non-high-income regions, the higher AAPC rise in AA lifetime risk calls for recognition that autoimmune diseases like AA may increasingly emerge during social development, which should prompt active improvement in prevention and treatment capabilities. These improvements should particularly focus on integrating and accounting for females’ greater biological and social susceptibility, especially in countries and regions with the highest AAPCs.

From age-specific features of sex disparity, females demonstrated increased AA lifetime risk across all age groups compared to males. Females experiencing an extended “vulnerability window” that spans three decades (ages 20–50) compared to males’ concentrated risk period (ages 20–30). Most strikingly, the finding that female-to-male relative risk reaches 4.89 among elderly populations in developed regions, represents a previously unrecognized epidemiological phenomenon with significant clinical implications. The overlap between peak female AA risk and reproductive years suggests hormonal modulation of disease susceptibility, while the persistence of elevated risk into post-menopausal years in high-SDI regions indicates that socioeconomic factors may override biological protection mechanisms [48, 49]. This extended exposure period likely creates cumulative effects that amplify lifetime risk, particularly when combined with sociocultural and socioeconomic factors. This life-course perspective reveals that AA in females may represent a “cumulative disadvantage” model where biological and sociocultural vulnerability, extended exposure periods, and socioeconomic amplification interact across decades to produce the observed disparities.

For SDI-dependent features of sex disparity, socioeconomically-boosted sex disparities suggest that AA burden reflects broader sex inequality, requiring public health responses beyond purely clinical approaches. The higher female-to-male relative risk in high-SDI regions suggests that socioeconomic development amplifies rather than reduces sex disparities—as societies develop, they may inadvertently create environments that maximize the expression of female biological and sociocultural vulnerability while providing insufficient protection against social risk factors. This calls for comprehensive female AA protection strategies that should include thorough investigation of development-associated risk factors, including urbanization effects, and healthcare system improvements with enhanced coverage for elderly females.

Moving forward, our findings generate several critical research priorities. Mechanistic studies should investigate the biological-social interaction model through longitudinal studies examining how social determinants modulate hormonal and immune pathways, development-associated risk amplification mechanisms including urbanization effects and psychosocial stress features, and protective factors in regions with lower-than-expected female risk. Intervention research should focus on life-course intervention models targeting multiple risk factors across extended vulnerability windows, health system innovations for addressing elderly female AA burden in developed regions, and social determinant interventions addressing sex inequities. Policy research should examine the effectiveness of sex-responsive health policies in reducing AA disparities and international comparative studies of successful models for addressing development-associated health disparities.

Several study limitations warrant acknowledgment, including reliance on GBD modeling assumptions, potential regional variations in diagnostic practices, and inability to fully account for genetic and environmental factors. Our study relied primarily on diagnostic codes rather than gold-standard dermatological examination and histopathological confirmation, which may have introduced diagnostic misclassification bias. Additionally, SDI and concentration indices may not fully capture healthcare inequities compared to more specific metrics such as Human Resources for Health, Universal Health Coverage, and Healthcare Access and Quality Index. However, the consistency of features across multiple analytical approaches and the biological plausibility of findings supports the robustness of our conclusions.

## Conclusion

Our analysis reveals pronounced sex disparities in global AA lifetime risk: consistently higher risk in females across regions and time periods, prolonged age-related risk window with slower decline in females, and greater risk elevation in females with increasing SDI levels.

## Supplementary Information

Below is the link to the electronic supplementary material.

Supplementary Material 1

Supplementary Material 2

Supplementary Material 3

Supplementary Material 4

Supplementary Material 5

Supplementary Material 6

Supplementary Material 7

## Data Availability

Further information and requests for data may be directed to and will be fulfilled by the Lead Contact: Prof. Wenhui Wang (wwh0608@126.com).
