# Supplementary material for: Global sex disparities in lifetime risk of alopecia areata: a systematic analysis from the global burden of disease study, 1990 to 2021
Source: Biol Sex Differ. 2025 Sep 24;16:68. doi: 10.1186/s13293-025-00749-w (PMC12462261; doi:10.1186/s13293-025-00749-w)
Supplement: Supplementary file 1 — Supplementary Material 1 [file 13293_2025_749_MOESM1_ESM.docx]

**Supplementary Information**

**Global Sex Disparities in Lifetime Risk of Alopecia Areata: A Systematic Analysis from the Global Burden of Disease Study, 1990 to 2021**

**Table Titles:**

**Table S1.** Global and sex-specific AA lifetime risk with corresponding female-to-male ratios, 1990-2021

**Table S2.** Projected global AA lifetime risk by sex with female-to-male ratios

**Table S3.** AAPC in AA lifetime risk stratified by geographic region and sex

**Table S4.** Age-specific AA lifetime risk stratified by sex, 2021

**Table S5.** AA lifetime risk stratified by SDI levels and sex with female-to-male ratios, 1990-2021

**Table S6.** Temporal trends in concentration indices for AA lifetime risk by sex and SDI with female-to-male ratios, 1990-2021

**
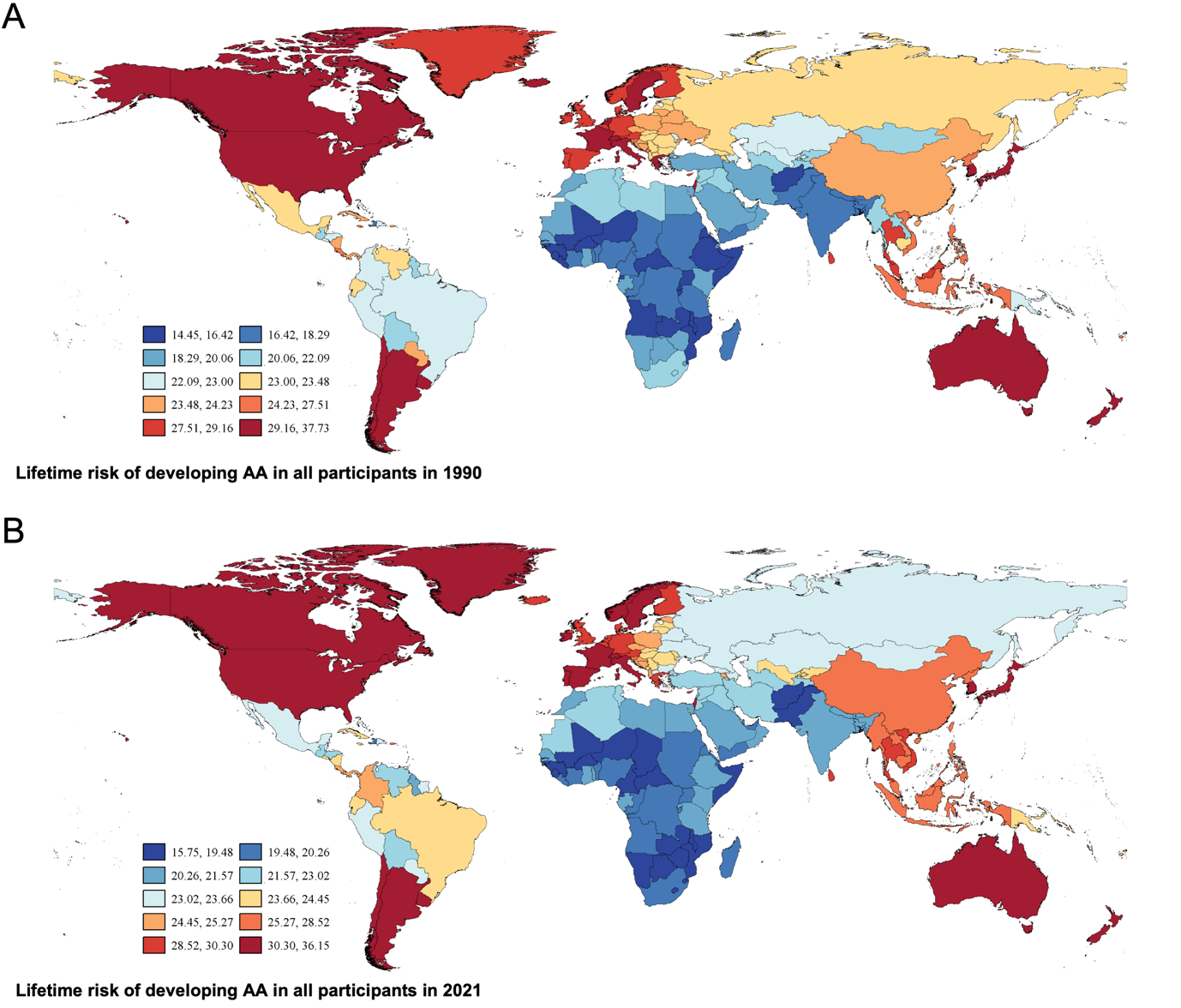
**

**Figure S1.** Global distribution of lifetime risk of all participants in 1990 (A) and 2021 (B)

**
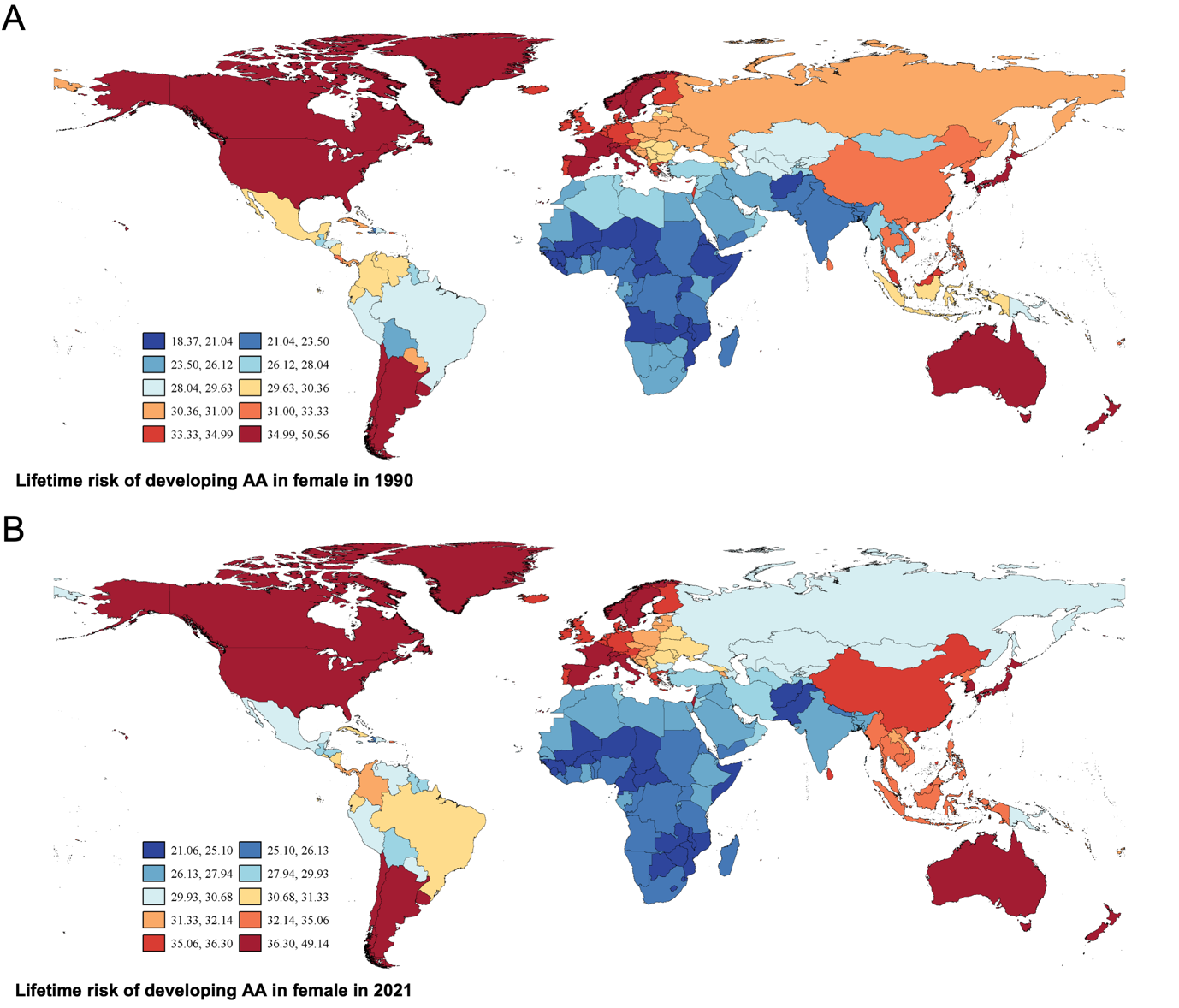
**

**Figure S2.** Global distribution of lifetime risk of female in 1990 (A) and 2021 (B)


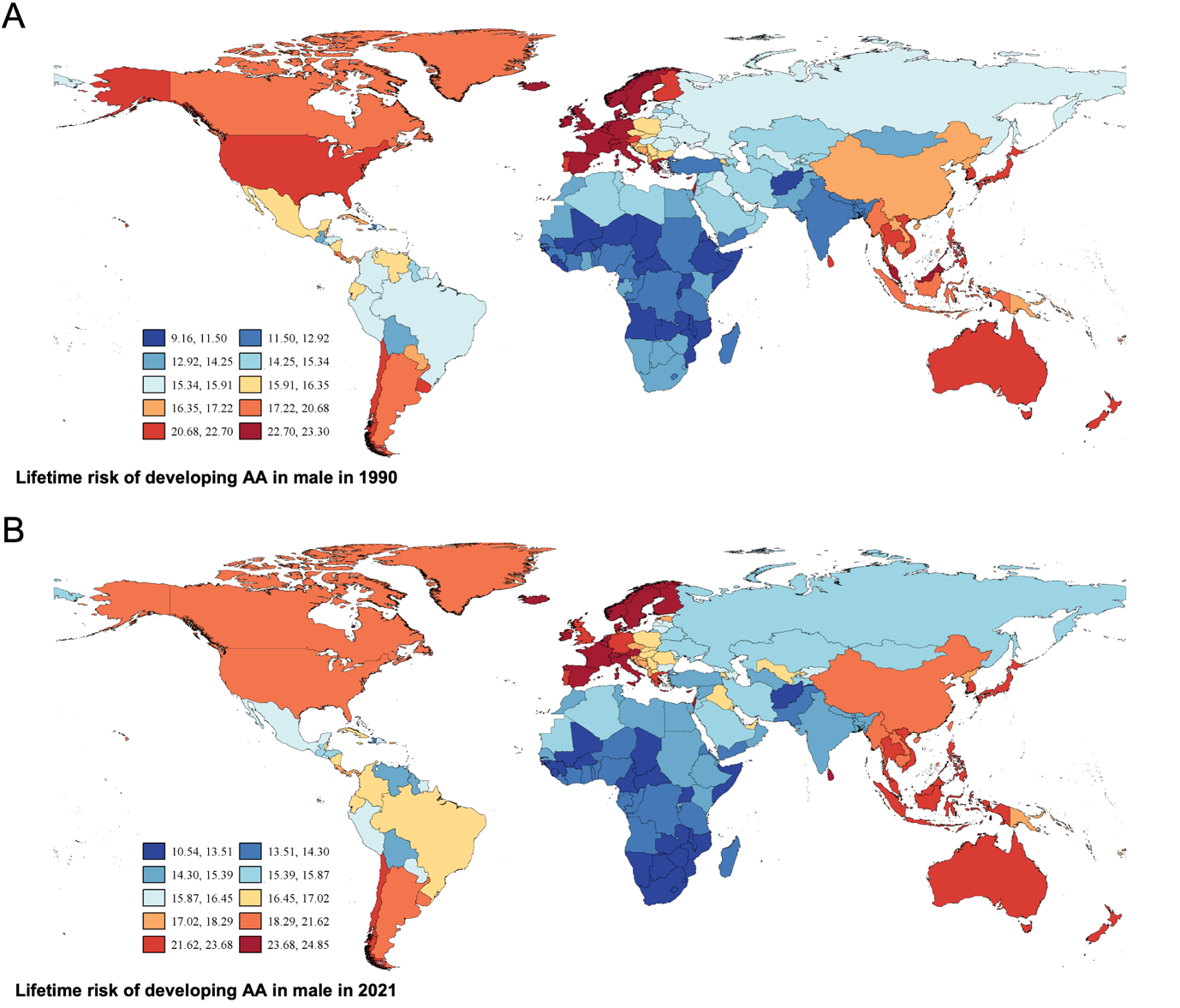


**Figure S3.** Global distribution of lifetime risk of male in 1990 (A) and 2021 (B)


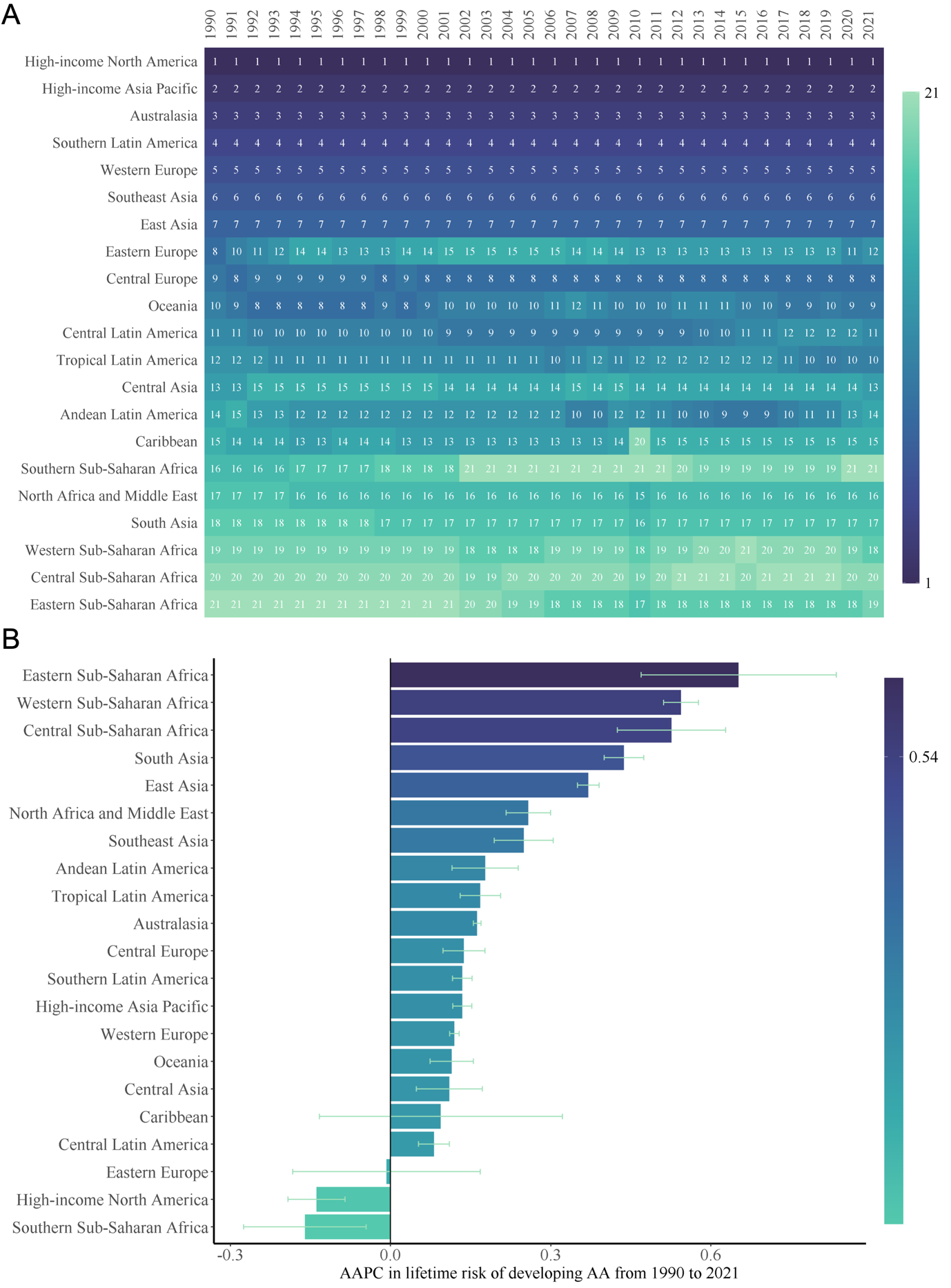


**Figure S4.** Regional temporal trends in overall lifetime risk (A) and AAPC (B), 1990-2021

**
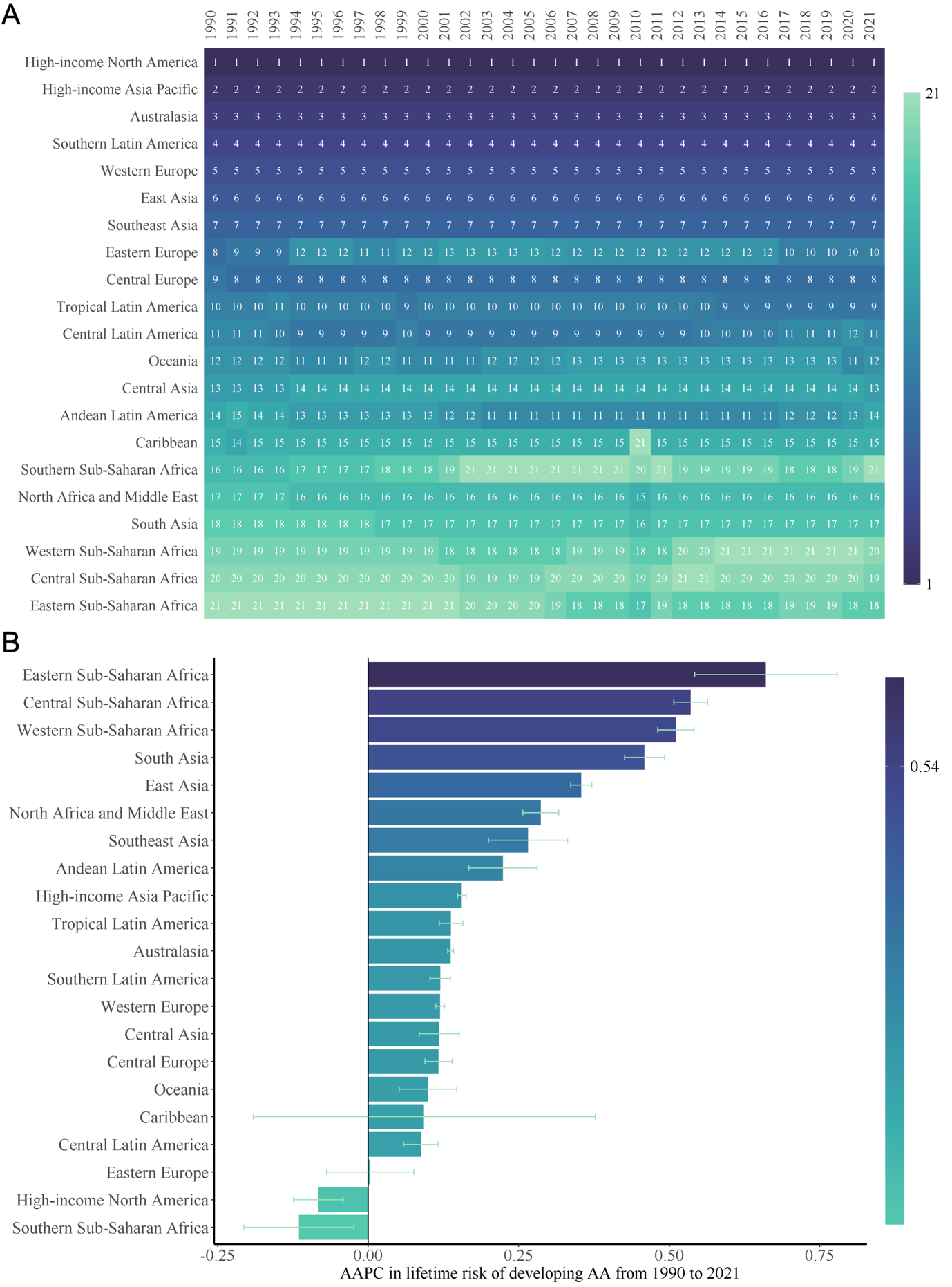
**

**Figure S5.** Regional temporal trends in female lifetime risk (A) and AAPC (B), 1990-2021


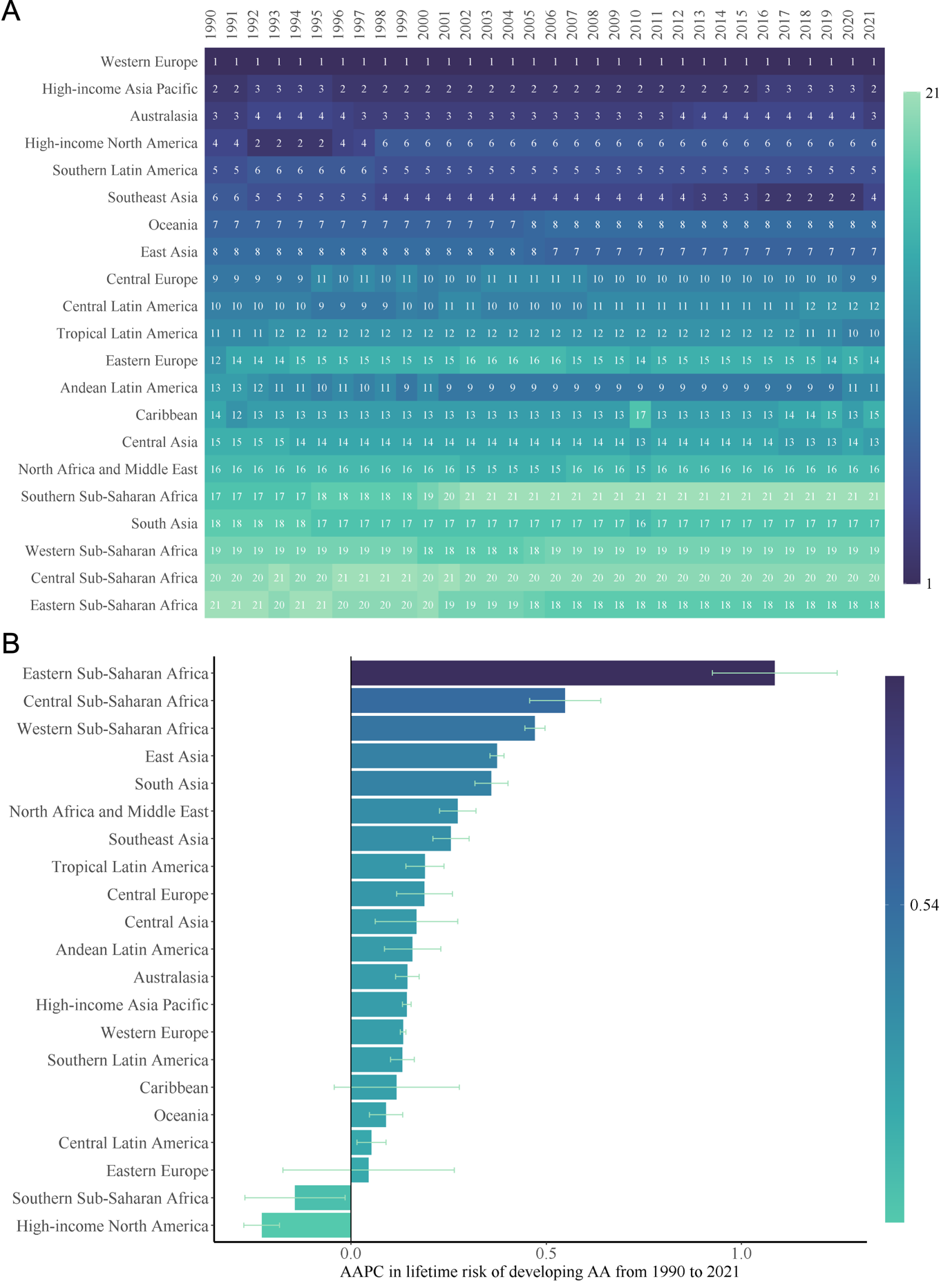


**Figure S6.** Regional temporal trends in male lifetime risk (A) and AAPC (B), 1990-2021


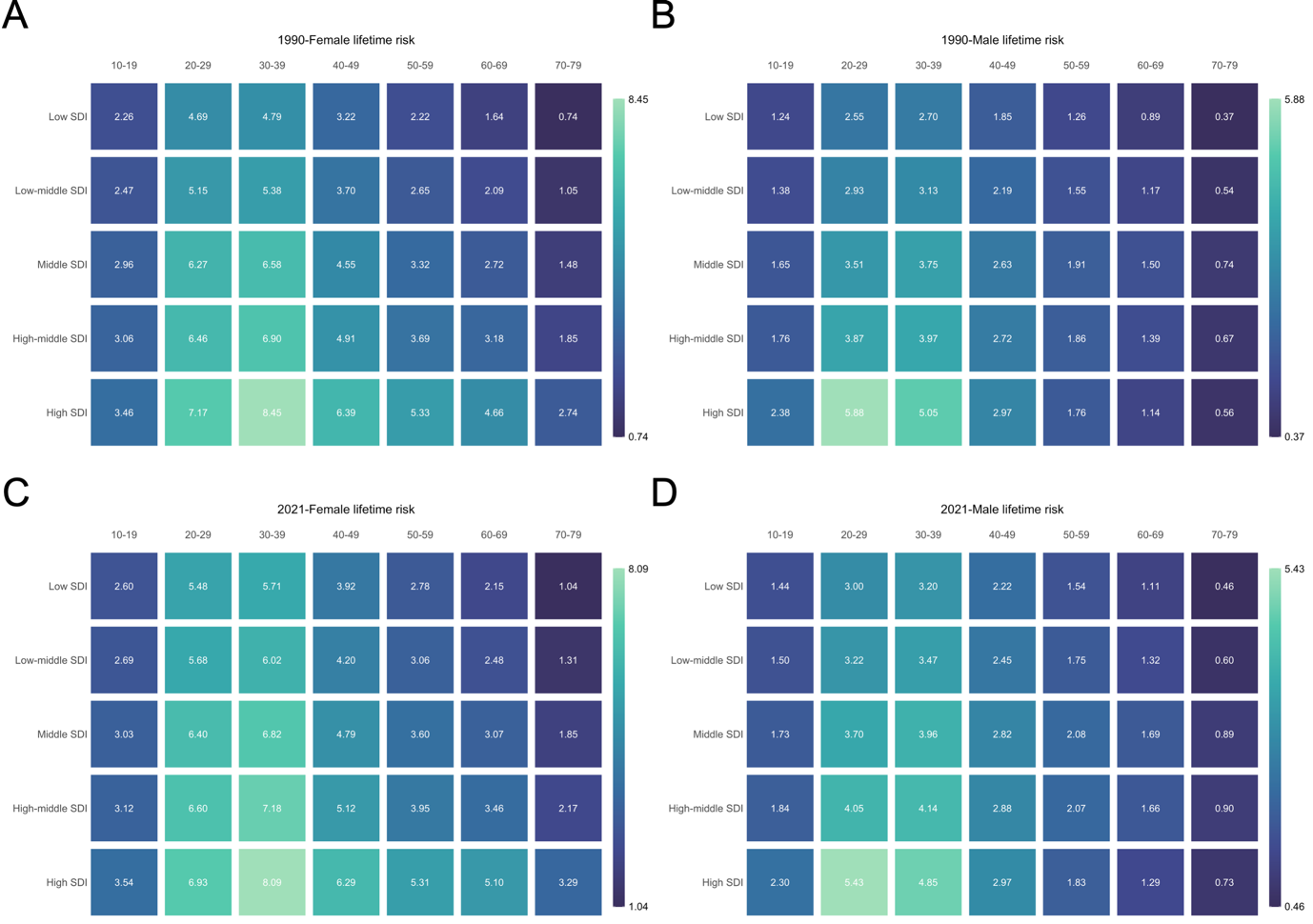


**Figure S7.** Age-stratified analysis of AA lifetime risk by gender and SDI levels in 1990 and 2021
